# Post-vaccination Monitoring to Assess Foot-and-Mouth Disease Immunity at Population Level in Korea

**DOI:** 10.3389/fvets.2021.673820

**Published:** 2021-08-04

**Authors:** Mi-Young Park, You Jin Han, Eun-Jin Choi, HeeYeon Kim, Rokeya Pervin, Wonseok Shin, Doheon Kwon, Jae Myoung Kim, Hyun Mi Pyo

**Affiliations:** Foot and Mouth Disease Diagnostic Division, Animal and Plant Quarantine Agency, Gimcheon-si, South Korea

**Keywords:** foot-and-mouth disease virus, population immunity, mass vaccination, vaccination campaign, post-vaccination monitoring

## Abstract

In South Korea, domestic cattle, pigs, and goats were subjected to mandatory foot-and-mouth disease (FMD) vaccination and year-round serosurveillance since 2011. In 2020, approximately USD 95 million was spent solely for FMD vaccine purchase for 59 million livestock, and 1.25 million samples were tested to estimate the population immunity and demonstrate the absence of virus circulation. As the FMD vaccination program was revised in 2018, the post-vaccination monitoring (PVM) was designed to evaluate the effectiveness of the vaccine program of three vaccines approved for routine use. To this end, monitoring post-vaccination immunity has been conducted by collecting 35,626 serum samples at 28 days post-vaccination following regular national vaccinations, which were carried out in April and in October in 2020. The design of the serological test for PVM was specially targeted at particular livestock groups, including dairy cattle, goats, and beef cattle aged 6–12 months, which were generally estimated to have a low expected seroprevalence. The risk factors had also been identified, considering the increased likelihood of infection in a particular location, herd size, and husbandry system applied in a targeted sample collection. Serum sample collection and SP-O and NSP antibody tests were performed by local veterinary laboratories using commercially available ELISAs. The current FMD vaccination program, which was performed twice a year following the regimen of primary vaccination and boost, resulted in over 80% population immunity. The seroprevalence monitored after the vaccination in fall was higher than the one studied in spring except in pigs. It was demonstrated that the seroprevalence of risk-based targeted samples ranged from 93.8 to 100% in cattle, 63.2 to 100% in pigs, and 20.0 to 100% in goats. Of note is the area near the North Korean borders which showed a relatively low seroprevalence among the targeted regions, and no NSP sero-positive reactor was detected in this region. When subpopulation immunity at the individual level was assessed, the seroprevalence in young cattle stock was slightly lower (95.8%) than that of adults (98.4%). In conclusion, the FMD vaccination campaign has been successfully implemented in Korea, and the PVM can be a supplementary program for massive routine surveillance in terms of providing timely information needed both to estimate population immunity and to properly target “risk-based surveillance.”

## Introduction

Post-vaccination monitoring (PVM) to evaluate the performance of vaccination regimens and program is essential for those countries embarking in vaccine-based foot-and-mouth disease (FMD) control policy (1–6). Especially in South Korea, 90% of the total budget, which was worth USD 98 million in 2020, assigned for FMD management is spent for vaccine purchase and vaccination in practice. PVM is important to evaluate the effectiveness of vaccines and vaccination program and plan for a future policy (7).

After experiencing a devastating FMD outbreak in 2010, a mandatory nationwide FMD vaccination for cattle, pigs, and goats was initiated to control the disease (8). Along with the implementation of FMD vaccines, a massive year-round serological surveillance program has been launched in 2011 to search the evidence of FMD virus (FMDV) circulation and evaluate population immunity. However, FMD was prevalent until 2016, and another large-scale FMD outbreak occurred in 2014–2015 (9–14) which prompted the introduction of diverse serotype O vaccine strains besides O1 Manisa. In 2017, a comprehensive biannual vaccination program for cattle and goats and post-vaccination sero-monitoring, in addition to year-round serosurveillance, were launched (10, 11, 15). The current vaccination regimen was adopted after the serotype A FMD outbreak in porcine in the spring of 2018. Presently, all susceptible livestock were vaccinated with oil-adjuvant inactivated vaccines, containing serotype O and A antigens, following a prime and boost inoculation schedule. As mentioned, the massive routine FMD serosurveillance, abiding by the national year-round surveillance program, provides valuable information on FMDV circulation in the field and population immunity by vaccination (16, 17). In this regard, 637,292 and 637,593 of serum samples were subjected to SP and NSP antibody ELISA, respectively, in 2020 (the national serosurveillance monthly report is available to the public at www.qia.go.kr). However, further information was required to evaluate the current vaccination regimens and program implemented at the end of 2018 as well as the success of the vaccination campaign. Hence, the sero-monitoring post-vaccination in 2020 was designed and applied to assess the impact of the current vaccination regimen and program by estimating vaccine-induced herd immunity at the population and subpopulation levels. In addition, the collected serosurveillance data were further analyzed at various subpopulation levels to evaluate the vaccine-induced immunity in high-risk groups.

## Materials and Methods

### Demographic Distribution of Livestock and FMD Surveillance System in South Korea

South Korea is comprised of eight cities and nine provinces. The demographics of cloven-hoofed livestock such as cattle, pigs, and goats are described in Supplementary Figure 1. The Ministry of Agriculture, Food, and Rural Affairs (MAFRA) supervises the national FMD vaccination and serosurveillance program, with the technical support of the Animal and Plant Quarantine Agency (APQA). The APQA plans for the national surveillance program and post-vaccination monitoring. Then, there are 46 regional veterinary services with trained veterinarians who conduct the sample collection and FMD diagnostic tests. An established vaccination registration system (Korea Animal Health Integrated system, KAHIS) to monitor vaccine distribution and administration regularly is operated by MAFRA, APQA, and the regional veterinary services.

### Vaccine Policy, Vaccination Regimen and Program

All cattle, pigs, and goats are subjected to mandatory FMD vaccination by The Act on the Prevention of Contagious Animal Diseases, and the public is notified regarding FMD vaccination, clinical examination, and retention of immunization. Farm owners should also keep the record of FMD vaccination and carry the certificate of FMD vaccination to present on demand during the movement of domestic animals for trade and slaughter. There is a heavy fine to be imposed for non-compliance.

Since October 2018, three FMD vaccines, oil-adjuvanted and containing inactivated serotype O and A antigens, were used for immunization in the field. The vaccine strains varied by the manufacturer, yet all three vaccines were equal or greater than three protective doses of 50% (PD_50_). The calf and young goats receive the primary vaccination at 2–4 months old and the boost injection at 4 weeks later. The piglets were vaccinated at 8–12 weeks old and received a boost injection at 4 weeks later. After the first two-dose FMD vaccination, all animals were vaccinated every 6 months. The injection dose is 2 ml for cattle and pigs and 1 ml for small ruminants. The vaccines are inoculated intramuscularly, and the neck and ham are the recommended injection sites. Further details of the FMD vaccines mentioned in this article are presented in Supplementary Table 1.

### Regular Biannual National Vaccination

Scheduled comprehensive national FMD vaccination campaigns are carried out, targeting all cattle and goats, in April and October considering the seasonal risk factor and 6-month interval of boost vaccination. Pig farms are generally excluded from the campaign schedule as they followed their own vaccination schedules optimized by the condition of each farm. In 2020, FMD vaccination was conducted from April 1 to May 28 (first round) and from October 5 to November 13 (second round). During this period, 3.5 million cattle and 525,926 goats were vaccinated (Table 1). The actual immunization of smallholdings with <50 cows was performed by a public veterinarian. In case of large-scale cattle farms, goat farms, and all pig operations, the farm owners were responsible for the vaccination on their own.

**Table 1:** Numbers of vaccinated animals and farms during the national vaccination in 2020 and the serum samples collected for post-vaccination monitoring.

**Species**	**No. of vaccinated farms**	**No. of vaccinated animals**	**Number of samples collected for post-vaccination monitoring**							
			**April**		**May^a^**		**October**		**November^a^**	
			**Farm**	**Animal**	**Farm**	**Animal**	**Farm**	**Animal**	**Farm**	**Animal**
Cattle	103,617	3,528,914	4,448	24,736	2,075	10,360	824	8,951	2,030	10,164
Pigs	5,122	12,646,088	1,911	33,582	-	-	2169	41,154	467	8,254
Goats	14,940	525,926	60	310	310	1,550	78	442	254	1,314
Total	123,679	16,700,928	6,419	58,628	2,385	11,910	3,071	50,547	2,751	19,732

a *May and November indicate 1 month after the first and second round of vaccination, respectively*.

### Supplementary Vaccination in a High-Risk Area

In addition to the nationwide systemic mass vaccination, supplementary vaccination to beef up the population immunity in high-risk regions was given in late September till early October of 2020. Indeed these high-risk regions or farms fall in one of the following categories: (i) regions where the NSP antibody-positive reactors in 2019 were detected, (ii) regions where the high-density pig operation complex with a previous history of FMD outbreak were located, (iii) farms located near the border to North Korea, and (iv) farms with a recent history of penalty imposition due to low herd immunity. The geographical distribution of these high-risk regions or farms was depicted in Supplementary Figure 2. A total of 1.02 million pigs from 530 farms, 204,844 cattle from 3,681 farms, and 20,396 goats from 493 farms were vaccinated in these regions. The trivalent vaccine, containing O, A, and Asia 1 antigens, was used in high-risk areas (Supplementary Table 1).

### Sample Collection

In May and November, a total of 31,642 blood samples were collected from cattle, goat, and pig farms by the 46 regional veterinary staff in cities and provinces. These samples were collected 1 month after the biannual vaccinations, which allowed us to evaluate the comprehensive national FMD vaccination (Supplementary Table 2).

The size of the farms for sample collection was estimated using a two-stage cluster sampling design based on the following parameters as described elsewhere (18): 95% confidence, 5% precision, and 80% expected sero-prevalence. Then, individual farms were selected randomly using simple random sampling. In addition, the sample size for sero-monitoring post-vaccination was determined by applying a weighting factor to the target subpopulation, which was 6–12-month-old beef cattle, dairy cattle, and animals in large-scale farms showing a low herd immunity. A total of 3,984 samples from the targeted subpopulation were also collected at 1 month post-supplementary vaccination in the high-risk areas mentioned above. The actual blood collection and visual inspection were conducted by the local veterinary staff and Livestock Health Control Association (LHCA), a public organization funded by the government. APQA releases the guidelines for the number of blood samples per farm and the age criteria for animal selection. In case of cattle and goat farms, five animals from a farm were randomly selected for sampling. For pig farms, 16 animals from a farm were subjected to blood sampling. Age-stratified sampling scheme was applied to cattle farms, with at least two samples from beef cattle aged 6–12 months that must be included among five samples. During blood sampling, the veterinary staff conducted a clinical examination concomitantly.

### Serological Tests

Serological tests were performed by the 46 regional veterinary laboratories in cities and provinces using commercially available ELISAs under the supervision of the central laboratory, APQA. Sera were assessed for vaccine-induced antibodies using three commercial type O SP antibody ELISAs: PrioCHECK^TM^ FMDV Type O Ab strip kit (Thermo Fisher Scientific), VDPro® FMDV Type O Ab b-ELISA (Median Diagnostics, South Korea), and BIONOTE FMD Type O Ab ELISA (BIONOTE Inc., South Korea). To identify the FMD virus infection, two commercial NSP antibody ELISAs—VDPro® FMDV NSP Ab ELISA (Median Diagnostics, South Korea) and BIONOTE FMD NSP Ab ELISA (BIONOTE Inc., South Korea)—were used according to the manufacturer's instruction. To increase the diagnostic specificity, a positive result in both NSP ELISAs was considered as NSP antibody-positive (19). Further details on the ELISA kits used in this study are presented in Supplementary Tables 3, 4.

### Statistical Analysis and Data Interpretation

R software (www.r-project.org) and EXCEL were used to compile the ELISA results. To evaluate the effectiveness of the vaccine program, population immunity surveyed by sero-monitoring post-vaccination was compared to the national serosurveillance data collected in April and October, which were considered to reflect population immunity before the regular biannual national vaccination (the national surveillance data is open to the public at www.qia.go.kr). A comparison by breeds (dairy and beef cattle), herd size (large-scale and smallholdings), and age criteria (6–12 months and over 1 year old in cattle, fattening, and breeding in pigs) was further analyzed to identify the risk factors. Paired *t*-test was performed, and *p* < 0.05 was considered statistically significant. For the purpose of this study, herd immunity was defined as seroprevalence with 95% confidence interval (CI) estimated for FMDV serotype O.

## Results

### Evaluation of Regular FMD Vaccination in 2020

**Figure 1:**
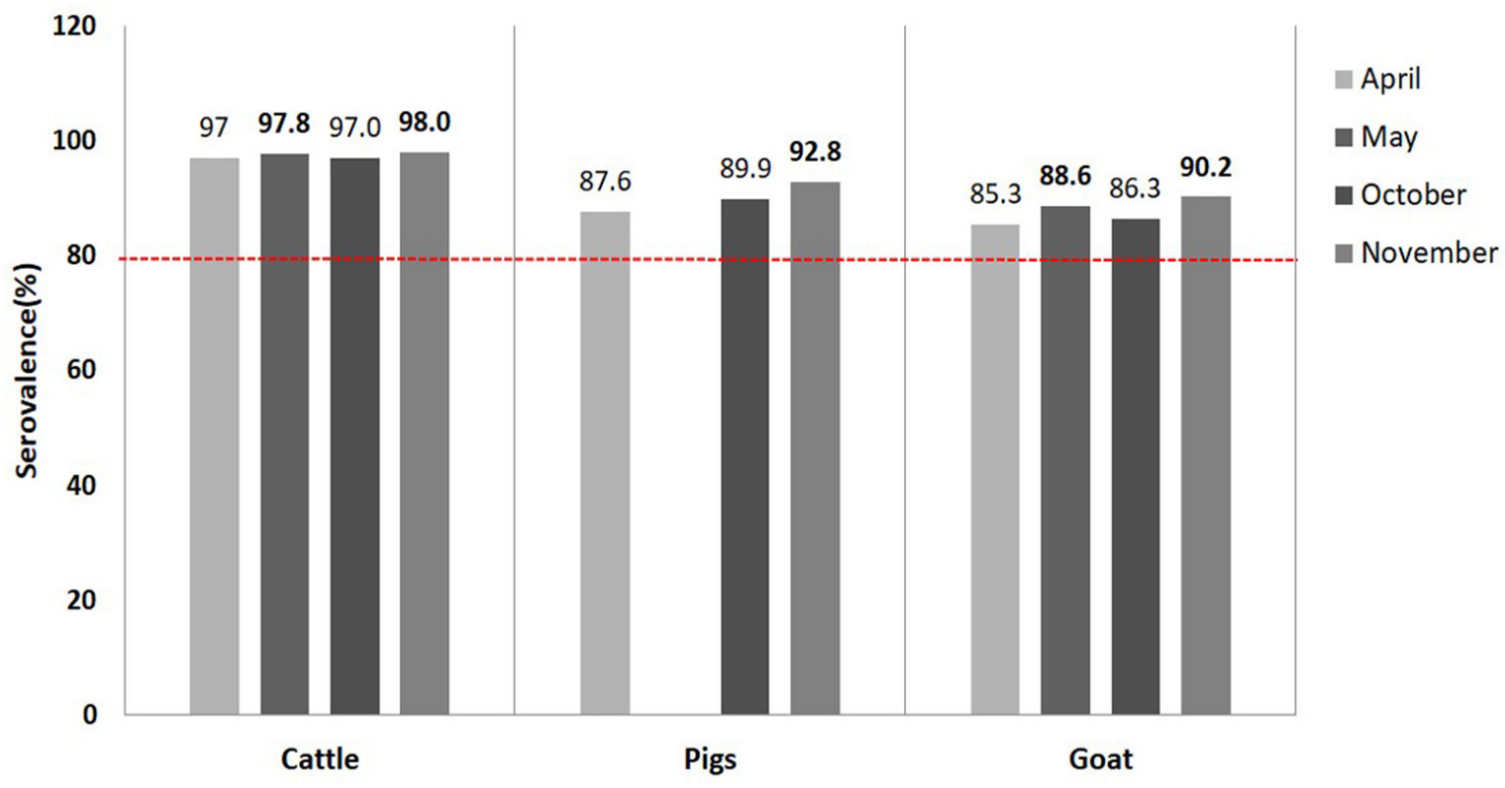
Vaccine-induced population immunity of livestock in 2020.

According to MAFRA, 59.4 million doses of FMD vaccines, worth USD 95 million, were released in 2020 to vaccinate domestic animals. The nationwide FMD vaccination was executed following the national FMD vaccination program in April and October. There was a total of 31,642 serum samples collected from 2,385 farms in nine provinces across the country at 1 month after the first round and second round of vaccination in May and November, respectively. The results of the clinical examination of the presence of FMD symptoms, conducted during blood collection, and NSP antibody ELISA indicated that there was no infection in the field (unpublished data). The species-dependent seroprevalence against serotype O was higher than 80% in all species tested after the mandatory scheduled vaccination (Figure 1). These results demonstrated that the scheduled systemic vaccination was effective to build up herd immunity. In detail, the seroprevalence by species studied after the second round of vaccination in November ranged from 90.2 to 98.0%, while that after the first round of vaccination in May was 88.6 to 97.8%. The seroprevalence of cattle was over 97.0% at all times, while those in pigs and goats were enhanced from 87.6 to 92.8% and 85.3 to 90.2%, respectively. These results suggested that the biannual vaccination practice was effective to improve the vaccine-induced immunity in pig and goat population.

### Regional Seroprevalence of FMD Vaccination

The seroprevalence by provincial level is presented in Table 2. There was no difference in seroprevalence between the provinces, except the seroprevalence of goats in Gyeongsangnam-do (GN) (72.5–73.0%), ChungcheongNam-do (75.7%), and GyeoungGi-do (78.8%) provinces, where the seroprevalence was relatively low compared to the seroprevalence of the goat population in other provinces (85.3–90.2%). According to individual farm data, such low seroprevalence in those three provinces was due to the negligence of vaccination of a few goat farms in the region. However, this unreliable immune status was improved at 1 month after the second round of vaccination in November.

**Table 2:** Population immunity (95% CI) at the province level by species in 2020.

**Province**	**April**		**May**		**October**		**November**	
	**No. of farms tested**	**Seroprevalence (%)**	**No. of farms tested**	**Seroprevalence (%)**	**No. of farms tested**	**Seroprevalence (%)**	**No. of farms tested**	**Seroprevalence (%)**
**Cattle**								
GG	638	97.2 (96.6–97.7)	345	98.9 (98.4–99.4)	92	97.5 (96.2–98.8)	295	98.2 (97.5–98.9)
GW	215	97.0 (96.0–98.1)	151	97.6 (96.6–98.7)	139	96.3 (95.0–97.6)	145	98.1 (97.1–99.0)
CB	277	96.9 (96.1–97.8)	151	96.7 (95.0–98.4)	37	98.4 (96.6–100)	129	98.8 (97.9–99.6)
CN	355	97.9 (97.0–98.8)	266	98.6 (98.0–99.3)	38	96.5 (94.1–98.9)	291	98.1 (97.3–98.8)
JB	617	97.2 (96.7–97.8)	251	98.5 (97.8–99.1)	96	96.0 (93.6–98.5)	224	98.2 (97.5–99.0)
JN	909	97.3 (96.8–97.8)	301	97.0 (96.0–98.0)	263	96.8 (95.6–98.0)	324	97.2 (96.3–98.2)
GB	914	97.0 (96.7–97.6)	395	97.5 (96.6–98.5)	101	99.5 (98.5–100)	390	98.5 (97.8–99.1)
GN	487	95.2 (94.2–96.2)	188	96.9 (95.4–98.4)	55	97.2 (95.4–99.0)	213	97.8 (97.0–98.7)
JJ	36	95.4 (92.3–98.4)	27	98.4 (96.5–100)	3	60.8 (0.0–100)	19	95.8 (91.0–100)
Total	4,448	97.0 (96.8–97.2)	2,075	97.8 (97.5–98.2)	824	97.0 (96.4–97.6)	2,030	98.0 (97.7–98.3)
**Pigs**								
GG	291	91.4 (89.6–93.3)			320	88.9 (86.9–91.0)	96	96.0 (94.5–97.8)
GW	54	82.9 (77.6–88.2)			78	88.9 (85.6–92.1)	18	86.8 (79.1–94.6)
CB	73	91.4 (87.8–95.1)			70	93.7 (91.0–96.4)	26	91.0 (85.5–96.5)
CN	374	92.1 (90.4–93.7)			570	93.8 (92.9–94.8)	95	96.4(94.4–98.4)
JB	198	90.3 (88.3–92.3)			316	87.0 (84.9–89.1)	59	95.3 (93.3–97.2)
JN	476	85.0 (83.3–86.7)			184	87.3 (84.6–90.0)	45	85.6 (80.1–91.2)
GB	240	83.3 (81.0–85.7)			170	86.0 (83.3–88.6)	57	88.7 (83.8–93.5)
GN	172	82.3 (78.8–85.7)			217	86.1 (83.8–88.5)	49	90.2 (86.6–93.9)
JJ	33	84.2 (77.0–91.5)			244	92.0 (90.1–93.8)	22	93.1 (89.5–96.6)
Total	1,911	87.6 (86.8–88.5)			2169	89.9 (89.1–90.5)	467	92.8 (91.6–94.0)
**Goats**								
GG	2	100 (100–100)	17	78.8 (69.6–88.0)	14	85.9 (76.5–95.3)	15	96.6 (92.6–100)
GW	3	86.7 (73.6–99.7)	16	91.3 (85.1–97.4)	21	84.8 (78.8–90.8)	16	90.0 (83.8–96.2)
CB	5	88 (72.3–100)	40	90.5 (86.1–94.9)	7	91.4 (79.8–100)	33	86.7 (81.6–91.7)
CN	8	87.5 (77.2–97.8)	35	93.7 (89.9–97.6)	7	75.7 (54.0–97.5)	31	89.7 (85.7–93.7)
JB	7	91.4 (83.5–99.4)	48	95.8 (93.0–98.7)	3	86.7 (60.5–100)	32	97.5 (95.2–99.8)
JN	8	97.5 (92.6–100)	60	90.3 (85.8–94.8)	12	90.0 (81.0–99.0)	46	90.4 (86.1–94.8)
GB	8	90.0 (82.6–97.4)	44	90.9 (87.9–93.9)	–	–	36	92.2 (86.1–98.3)
GN	19	72.5 (58.1–86.9)	46	73.0 (64.1–82.0)	14	88.6 (79.6–97.5)	43	83.3 (75.9–90.6)
JJ	-	-	4	100 (100–100)	-	-	2	100 (100–100)
Total	60	85.3 (79.7–90.9)	310	88.6 (86.5–90.7)	78	86.3 (82.4–90.2)	254	90.2 (88.2–92.2)

*GG, GyeoungGi-do; GW, GangWon-do; CB, ChungcheongBuk-do; CN, ChungcheongNam-do; JB, JeollaBuk-do; JN, JeollaNam-do; GB, GyeongsangBuk-do; GN, Gyeongsangnam-do; JJ, Jeju-do*.

### Assessment of Herd Immunity at the Farm Level

**Figure 2:**
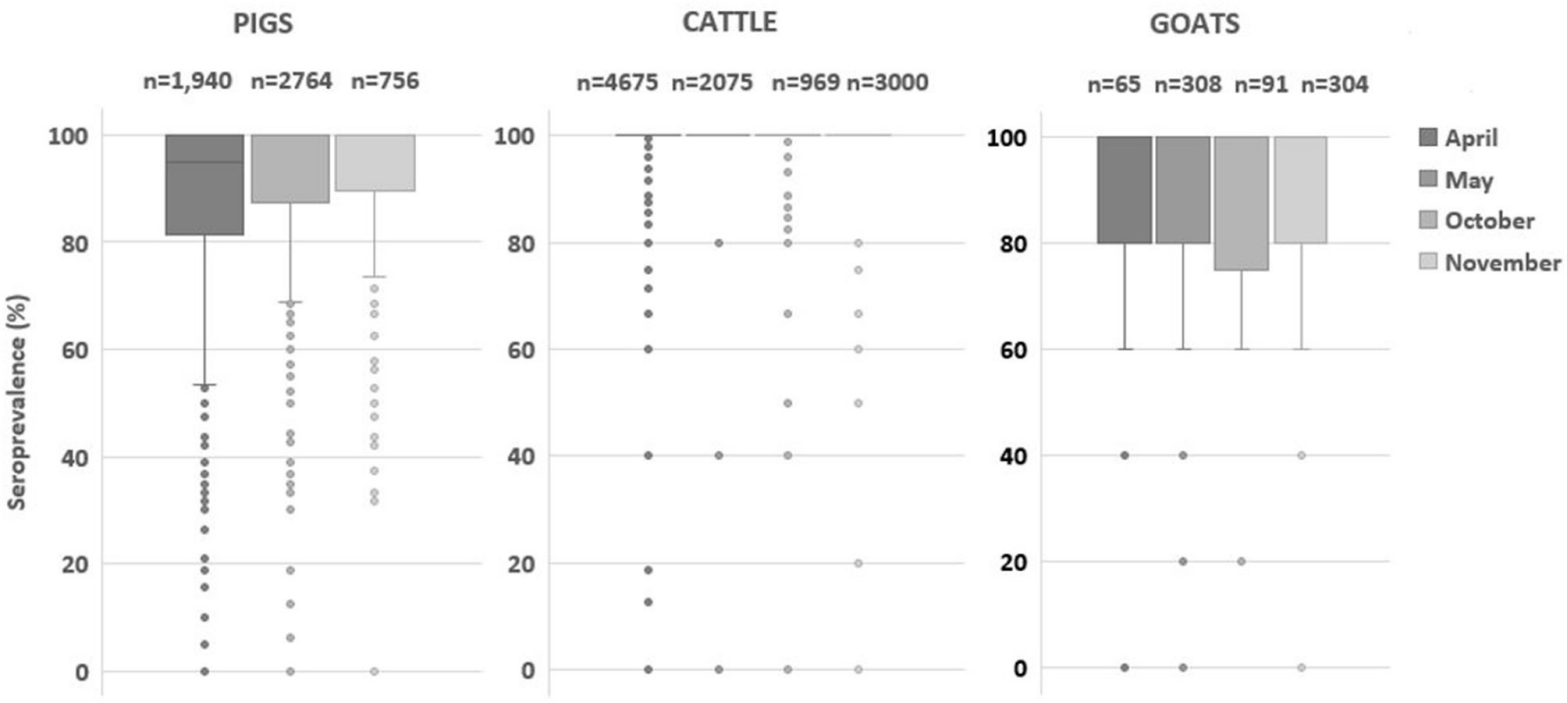
Comparison of herd immunity (%) estimated at the farm level by species. The box plot graph presents foot-and-mouse disease serotype O seroprevalence (%) of cattle, pigs and, goats in April and October (pre-biannual vaccination) and May and November (post-biannual vaccination). The box plot represents inter-quartile range, and the horizontal line is the median value.

Provided that vaccine is effective to circulating virus, a farm-based herd immunity of over 80% is expected to stop viral transmission (1, 2, 20, 21). The majority of cattle farms maintained adequate immune status as presented in Figure 2, and the farms with <80% herd immunity were <1% of the total cattle farms (Table 3). Such results implied that FMD vaccination was well-conducted in cattle compared to that in other livestock. In the case of pig farm, the proportion of farms with <80% immunity steadily decreased from 24.0 to 13.1% (Table 3). Similarly, the proportion of goat farms with <80% immunity rapidly dropped from 25.3 to 10.2% after the second round of vaccination (Table 3). These results suggest that the current biannual vaccination program was effective to increase and maintain the level of herd immunity in these species.

**Table 3:** Proportion of the farms with <80% herd immunity.

**Species**	**Total no. of farms**	**April**		**May**		**October**		**November**	
		**No. of farms tested**	**Proportion^a^** **(no. of farms)**	**No. of farms tested**	**Proportion^a^** **(no. of farms)**	**No. of farms tested**	**Proportion^a^** **(no. of farms)**	**No. of farms tested**	**Proportion^a^** **(no. of farms)**
Cattle	107,939	4,675	0.79 (37)	2,075	0.39 (8)	969	0.7 (7)	2,030	0.3 (7)
Goats	15,678	65	18.5 (12)	310	12.3(38)	91	25.3 (23)	254	10.2 (26)
Pigs	5,219	1,940	24.0 (465)	-	-	2,200	19.3 (425)	467	13.1 (61)

a *The proportion of farms with <80% herd immunity*.

### Age, Herd Size, and Regional Factors Affecting Subpopulation Immunity

The subpopulation immunity of cattle and pigs in different age groups is presented in Table 4. The seroprevalence of young calves was lower (88.8–96.2%) than that of cattle (97.6–98.7%) in the course of time (*p* < 0.05). A similar finding was observed in pigs as the population immunity of fattening pigs (86.7–91.7%) was significantly lower (*p* < 0.01) than that of the breeding pigs (94.2–97.0%). Nevertheless, the proportion of sero-positive cattle aged 6–12 months increased after the second round of vaccination from 88.8 to 95.5%. Similarly, in fattening pigs, the population immunity was considerably enhanced from 86.7 to 91.7% in November.

**Table 4:** Subpopulation immunity by age.

**Category**		**April**		**May**		**October**		**November**	
**Species**	**Age stratum**	**No. of samples**	**Prevalence (%)**	**No. of samples**	**Prevalence (%)**	**No. of samples**	**Prevalence (%)**	**No. of samples**	**Prevalence (%)**
Cattle	6–12 months	3,652	93.4	2,159	96.2	402	88.8	1,010	95.5
	>12 months	22,423	97.6	8,201	98.3	9,448	98.7	6,852	98.3
	*p* value		0.0452		0.0002		0.007		0.0038
Pigs	Fattening	30,702	86.7	-	-	37,944	89.0	6,868	91.7
	Breeding	3,443	94.2	-	-	3,805	95.8	1,386	97.0
	*p* value		0.0005	-	-		0.0000		0.0003

**Figure 3:**
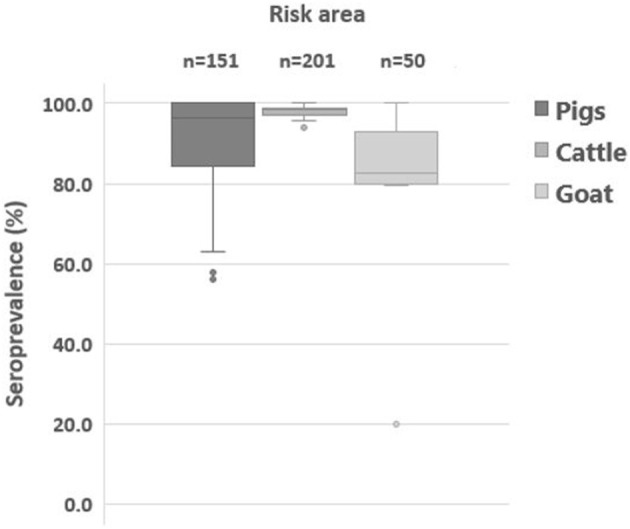
Distribution of herd immunity (%) estimated at a targeted farm in high-risk areas. Box plot graph presents foot-and-mouse disease serotype O seroprevalence (%) of cattle, pigs, and goats at 1 month after the supplementary vaccination in high-risk areas. The box plot represents inter-quartile range, and the horizontal line is the median value.

The subpopulation immunity of cattle categorized by herd size and breed is presented in Table 5. There was no difference in herd immunity by farm size (*P* > 0.05). The herd immunity of large-scale cattle farms was between 96.7 and 98.9%. The herd immunity of smallholdings was similar to that of large-scale cattle farms (96.5 and 98.4%). However, herd immunity was different by breed. Compared to the herd immunity of beef farms, those of dairy farms were higher throughout the year except after the second round of vaccination in November (*p* = 0.4118). Data on seroprevalence after the supplementary vaccination in high-risk regions are summarized in Table 6. The population immunity of cattle in high-risk areas was 96.2–98.7%, that of pig was 63.2–99.0%, and that of goats was 72.2–85.3%. However, the herd immunity of districts where the high-risk regions belonged to was 93.8–100% in cattle, 63.2–100% in pigs, and 20.0–100% in goats (Figure 3). In addition, we noted that those located near the North Korea border showed a low seroprevalence among the high-risk areas.

**Table 5:** Subpopulation immunity (95% CI) by herd size and breed.

**Category**		**Total no. of farms**	**April**			**May**			**October**			**November**		
			**Farm**	**Sample**	**Sero-prevalence (%)**	**Farm**	**Sample**	**Sero-prevalence (%)**	**Farm**	**Sample**	**Sero-prevalence (%)**	**Farm**	**Sample**	**Sero-prevalence (%)**
Herd size	Large scale	23,485	3,417	19,443	96.7 (96.5–97.0)	1,640	8,205	97.7 (97.3–98.1)	369	6,761	97.6 (96.5–98.6)	766	3,837	98.0 (97.5–98.5)
	Smallholding	84,454	682	3,538	97.4 (96.9–98.0)	435	2,155	98.4 (97.9–98.9)	390	1,739	96.2 (95.3–97.0)	1,264	6,327	98.1 (97.7–98.4)
	*P* value				0.5258			0.0571			0.0724			0.8389
Breed	Beef cattle	102,458	4,099	22,981	96.9 (96.6–97.1)	1,653	8,250	97.6 (97.2–98.0)	759	8,500	96.9 (96.2–97.5)	1,573	7,862	97.9 (97.6–98.3)
	Dairy cattle	5,481	349	1,755	98.7 (98.2–99.3)	422	2,110	98.7 (98.3–99.2)	65	451	98.5 (97.2–99.8)	457	2,302	98.5 (98.0–99.0)
	*P* value				0.0018			0.0432			0.0426			0.4118

**Table 6:** Herd immunity (95% CI) in a high-risk area.

**Category^a^**	**Province**	**Cattle**					**Pigs**					**Goats**				
		**No. of districts**	**Total farms**	**Sample no**.		**Sero-prevalence (%)**	**No. of districts**	**Total farms**	**Sample no**.		**Sero-prevalence (%)**	**No. of districts**	**Total farms**	**Sample no**.		**Sero-prevalence (%)**
				**Farm**	**Animal**				**Farm**	**Animal**				**Farm**	**Animal**	
1	IC	1	18	18	90	98.9 (96.7–100)										
2	IC	2	622	30	150	98.7 (96.9–100)	1	1	1	19	63.2	2	132	13	63	79.0 (68.8–89.1)
	GG	4	1,403	72	358	98.3 (97.0–99.6)	6	245	70	1,302	86.5 (82.1–90.9)	4^b^	176	18	90	72.2 (53.2–91.3)
	GW	5	1,638	81	405	96.2 (94.3–98.1)	8	120	34	634	91.2 (87.2–95.1)	5	185	19	95	85.3 (79.4–91.1)
	Subtotal	11	3,663	183	913	97.4 (96.4–98.5)	15	366	105	1,955	87.8 (84.5–91.1)	11	493	50	248	78.9 (71.2–86.6)
3	CN						2	43	13	214	99.0 (90.9–100)					
	JB						1	40	10	160	98.1 (92.2–100)					
	Subtotal						3	83	23	374	98.6 (97.3–100)					
4	-						22	81	23	404	91.5 (85.4–97.7)					
Total		12	3,681	201	1,003	97.6 (96.6–98.5)	58	530	151	2,733	89.9 (87.4–92.5)	11	493	50	248	78.9 (71.2–86.6)

a *Farms were selected in these regions based on the risk factors of the following categories: (1) regions where the NSP antibody-positive reactors in 2019 were detected; (2) having farms located near the border to North Korea; (3) regions where the high-density pig operation complex with previous history of foot-and-mouth disease outbreak was located; and (4) farms with a recent history of penalty imposition due to low herd immunity*.

b *The seroprevalence in Paju, one of the four districts in GG, was 20.0% due to four goat farms with 0.0% herd immunity*.

*IC, InCheon; GG, GyeoungGi-do; GW, GangWon-do; CN, ChungcheongNam-do; JB, JeollaBuk-do*.

### Evaluation of the FMD Vaccination Regimen in Pigs

The population immunity in pigs progressively increased from 87.6 to 92.8% in 2020. Fattening pigs particularly showed a rapid increase of subpopulation immunity from 86.7% in May to 91.7% in November (Table 4). In addition, the proportion of farms with herd immunity below 80% steadily decreased from 24.0% in May to 13.1% in November (Table 3 and Figure 3). These results suggested that the current vaccination regimen, which is comprised of prime and boost immunization, is effective to induce a vaccine-derived antibody response in pigs. Similarly, a high level of seroprevalence of over 88% in cattle aged 6–12 months demonstrated the effectiveness of the current biannual vaccination program.

## Discussion

Massive vaccination, monitoring of post-vaccination immunity, and active and passive serosurveillance are important measures for the control of FMD (1–6, 24). Experiences from South America and Europe suggested that the implementation of a systematic vaccine policy can successfully eradicate FMDV (3, 22, 25). After the massive FMD outbreaks in 2010, South Korea initiated a mandatory nationwide vaccination and serosurveillance to estimate the overall population immunity either by previous infection or vaccination. In addition, post-vaccination monitoring was implemented to help in the impact assessment of the current vaccination program and in identifying the weakness of the vaccination campaign by estimation of population immunity at various categories in 2017 (1, 2, 22–26).

In the present study, data on post-vaccination sero-monitoring conducted in 2020 after two rounds of vaccination in May and November to monitor the immune status at population, district, farms, and subpopulation levels in Korea are presented. During the post-vaccination sero-monitoring, all serum samples were NSP antibody-negative in two NSP Ab ELISAs such that it substantiated the absence of virus circulation and transmission (27–29). In addition, this result implied that the population immunity was sorely derived from the vaccination.

The overall population immunity from April to November in 2020 was consistently higher than 80% in all the targeted species including pigs. To achieve over 80% herd immunity in pig and in under-1-year-old population, prime and boost vaccination regimens were implemented in late 2018. The sero-monitoring and national serosurveillance results showed that the seroprevalence in the pig population was over 80% regardless of time, and the proportion of farms with herd immunity under 80% was decreased in 2020. Such high population immunity in pig has significance considering that most of the pigs are subjected to FMD vaccination once during their lifetime, and the immune response to FMD vaccines was relatively weak and readily waned in porcine (30–33). Therefore, the results of our study suggest that the current vaccination regimen, which adopted priming at 8–12 weeks old and boosting in a month, was effective in the young age group of pigs as well as in other species. In many Asian countries where a major target for vaccination to FMDV is pigs, they strive to achieve a herd immunity of 80% or more (30–32). However, the evaluation of FMD vaccine performance is mainly emphasized on their use in cattle, so there are difficulties to collate data for optimizing FMD vaccination regimens in pigs. Therefore, it was very encouraging to reach almost 90% of immunity in pigs, an important domesticated livestock species in Korea.

Population immunity was further assessed in various subpopulations to identify the potential risk factors. There was no significant difference of immunity in cattle by herd size. However, there were slight differences in age group as adult cattle over 12 months old showed a higher seroprevalence compared to young stocks under 12 months old. Such observation may be due to the cumulative vaccination effect in adult cattle population. Similarly, except after the second round of vaccination in November, there was a slight difference in immunity between dairy and beef cattle. Nevertheless, the systematic biannual vaccination adhering to a synchronized schedule of vaccine administration led to improved vaccine coverage and resulted in a positive impact of vaccination campaigns. The Korean government also provides support, including subsidies for vaccine purchase, public veterinarian for vaccine inoculation, and educational campaign for farmers and stakeholders, which encouraged FMD vaccination.

In order to determine a high-risk region for additional supplementary vaccination, previous epidemiological and serosurveillance results were used (19). The high-risk regions were locations such as those where previous FMD outbreaks had occurred, the ones near the North Korea borders, areas where the farms have NSP sero-positive reactor or the ones with a record of such, and locations of farms with a recent history of penalty imposition due to non-compliance with the vaccination program. The seroprevalence of pigs and goats was slightly low in these regions. It was especially noted that few farms with low herd immunity were located near the North Korea border. However, there was no NSP antibody-positive case found in all the samples tested, indicating no circulation or transmission of FMDV. Supplementary vaccination and sero-monitoring post-vaccination in high-risk regions allowed the detection of farms and subpopulations with inadequate immunity and subsequent corrective actions by the government.

If properly planned and conducted, sero-monitoring post-vaccination could replace mass serosurveillance at a fraction of the resources and cost. In 2020, approximately USD 5 million was spent to purchase serological test kits, and 1.3 million serological samples were tested for routine serosurveillance. In addition, such a mass sampling requires the cooperation of farm owners, and it can be a necessary burden. As described, sero-monitoring post-vaccination was able to provide sufficient information for estimating herd immunity at various population levels and identifying risk factors. The generated data can also help to refine the vaccination regimens and programs and evaluate the vaccination campaign. Hence, post-vaccination sero-monitoring could substitute mass serosurveillance if budget and resources are scarce (7).

## Data Availability Statement

Publicly available datasets were analyzed in this study. This data can be found here: http://www.qia.go.kr/animal/prevent/listwebQiaCom.do?type=2_8ktyzl&amp.

## Author Contributions

M-YP conceived and designed the study and drafted the manuscript. YH, HK, and RP performed the serological tests and contributed to data arrangement. E-JC, WS, and DK collected and analyzed the serosurveillance data. E-JC and JK directed the project. HP reviewed and revised the manuscript. All authors approved the final version of the manuscript for publication.

## Conflict of Interest

The authors declare that the research was conducted in the absence of any commercial or financial relationships that could be construed as a potential conflict of interest.

## Publisher's Note

All claims expressed in this article are solely those of the authors and do not necessarily represent those of their affiliated organizations, or those of the publisher, the editors and the reviewers. Any product that may be evaluated in this article, or claim that may be made by its manufacturer, is not guaranteed or endorsed by the publisher.
