# Supplementary figures and images for: Post-vaccination Monitoring to Assess Foot-and-Mouth Disease Immunity at Population Level in Korea
Source: Front Vet Sci. 2021 Aug 4;8:673820. doi: 10.3389/fvets.2021.673820 (PMC8371437; doi:10.3389/fvets.2021.673820)

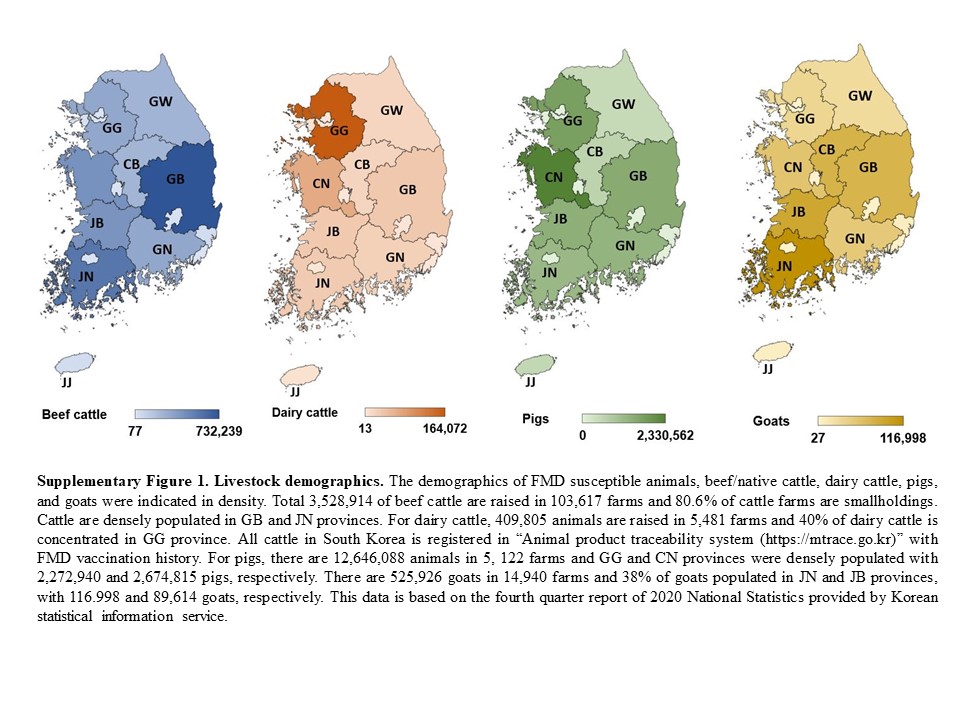

Supplement: Supplementary file 1 [file Image_1.jpg]

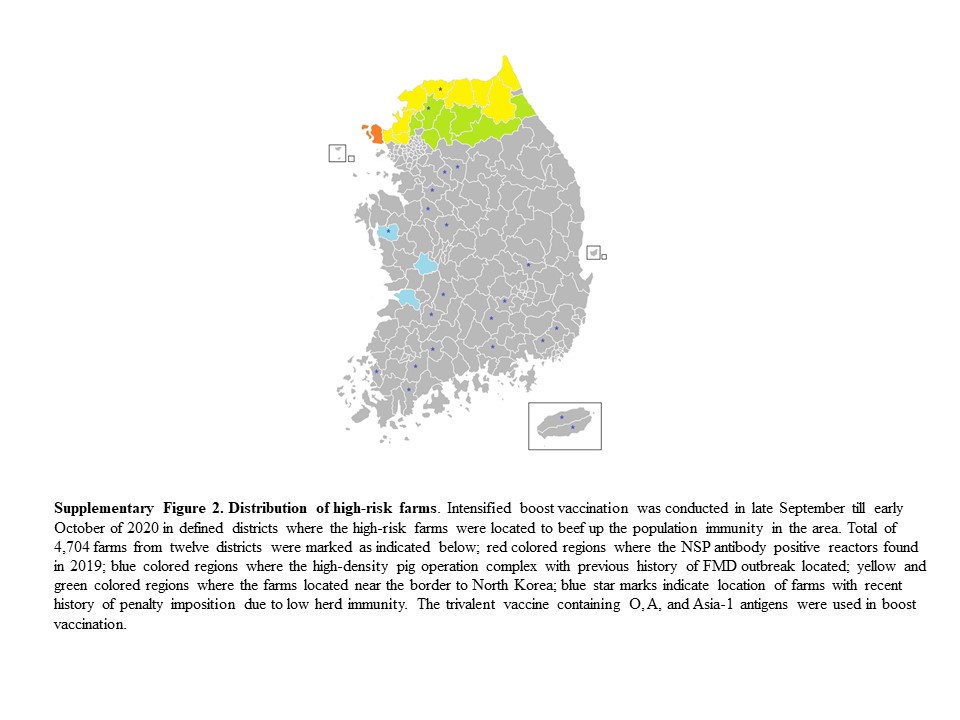

Supplement: Supplementary file 2 [file Image_2.jpg]
