# Supplementary material for: Post-vaccination Monitoring to Assess Foot-and-Mouth Disease Immunity at Population Level in Korea
Source: Front Vet Sci. 2021 Aug 4;8:673820. doi: 10.3389/fvets.2021.673820 (PMC8371437; doi:10.3389/fvets.2021.673820)
Supplement: Supplementary file 3 [file Data_Sheet_1.docx]

**Supplementary Table 1. Summary of FMD vaccines used in 2020**

| Product | Manufacturer | Serotypes | Virus Strains | Applicable species and vaccine schedules | Dose |
| --- | --- | --- | --- | --- | --- |
| AFTOPOR/ UK | Merial Animal Health Ltd. | O+A | O1 Manisa,  O 3039,  A22 Iraq  (≥ 3PD50) | Calf, Young goats: 1^st^ injection at two months of age, 2^nd^injection at 4wks later  Cattle: Revaccination 4∼7 months later  Piglet: 1^st^ injection at 8∼12wks of age, 2^nd^injection at 4wks later | Cattle, Pigs: 2ml per inj.  Goats, sheep: 1ml per inj. |
|  |  |  |  |  |  |
|  |  |  |  |  |  |
| ARRIAH / RUSSIA | FGBI | O+A | O Primorsky,  A Zabaikalsky  (≥ 6PD50) | Calf: 1^st^injection at four months of age, 2^nd^injection at 4wks later  Cattle: Revaccination 6 months later  Piglet, Sheep, Goats: 1^st^injection at 8∼12wks of age, 2^nd^injection at 4wks later |  |
|  | ARRIAH |  |  |  |  |
|  |  |  |  |  |  |
| ATOGEN / Argentina | Biogenesis | O+A | O1 Campos,  A24 Cruzeiro,  A2001 Argentina  (≥ 6PD50) | Calf, Goats: 1^st^injection at four months of age, 2^nd^injection 3~4wks later  Cattle: Revaccination 6 months later  Piglet: 1^st^injection at 8∼12wks of age, 2^nd^injection at 4wks later |  |
|  | BAGO |  |  |  |  |
|  |  |  |  |  |  |
| ARRIAH / RUSSIA | FGBI | O+A+Asia1 | O Primorsky,  A Zabaikalsky,  Asia1 Shamir | Calf: 1^st^injection at four months of age, 2^nd^ injection at 4wks later  Cattle: Revaccination 6 months later  Piglet, Sheep, Goats: 1^st^injection at 8∼12wks of age, 2^nd^injection at 4wks later |  |
|  | ARRIAH |  |  |  |  |
|  |  |  |  |  |  |

**Supplementary Table 2. Summary of sample collection by species and provinces**

|  |  |  |  | **Cattle** | | | | | | | |
| --- | --- | --- | --- | --- | --- | --- | --- | --- | --- | --- | --- |
| Province | Total districts in province | Total number of farms | Cattle  Population | Total number of sampled | | | | | | | |
|  |  |  |  | April | | **May** | | October | | **November** | |
|  |  |  |  | Farm | Animal | **Farm** | **Animal** | Farm | Animal | **Farm** | **Animal** |
| GG | 31 | 9,585 | 405,458 | 638 | 3,740 | **345** | **1,725** | 92 | 955 | **295** | **1,488** |
| GW | 18 | 7,893 | 264,974 | 215 | 1,048 | **151** | **735** | 139 | 1,126 | **145** | **725** |
| CB | 11 | 6,490 | 257,082 | 277 | 1,602 | **151** | **761** | 37 | 522 | **129** | **645** |
| CN | 15 | 14,185 | 439,856 | 355 | 1,915 | **266** | **1,335** | 38 | 166 | **291** | **1,457** |
| JB | 14 | 10,831 | 428,859 | 617 | 3,077 | **251** | **1,255** | 96 | 1,648 | **224** | **1,120** |
| JN | 22 | 18,973 | 546,100 | 909 | 5,533 | **301** | **1,505** | 263 | 2,452 | **324** | **1,619** |
| GB | 23 | 22,289 | 813,143 | 914 | 4,482 | **395** | **1,979** | 101 | 1,607 | **390** | **1,950** |
| GN | 18 | 12,462 | 333,731 | 487 | 2,617 | **188** | **940** | 55 | 434 | **213** | **1,065** |
| JJ | 2 | 909 | 39,711 | 36 | 722 | **27** | **125** | 3 | 41 | **19** | **95** |
| Total | 154 | 103,617 | 3,528,914 | 4,448 | 24,736 | **2,075** | **10,360** | 824 | 8,951 | **2,030** | **10,164** |

|  |  |  |  | **Pigs** | | | | | | | |
| --- | --- | --- | --- | --- | --- | --- | --- | --- | --- | --- | --- |
| Province | Total districts in province | Total number of farms | Pigs  Population | Total number of sampled | | | | | | | |
|  |  |  |  | April | | **May*** | | October | | **November** | |
|  |  |  |  | Farm | Animal | **Farm** | **Animal** | Farm | Animal | **Farm** | **Animal** |
| GG | 31 | 951 | 2,272,940 | 291 | 4,913 |  |  | 320 | 5527 | **96** | **1,661** |
| GW | 18 | 193 | 562,537 | 54 | 1,000 |  |  | 78 | 1,820 | **18** | **342** |
| CB | 11 | 295 | 707,863 | 73 | 1,331 |  |  | 70 | 1249 | **26** | **449** |
| CN | 15 | 1,090 | 2,674,815 | 374 | 6,601 |  |  | 570 | 11500 | **95** | **1,635** |
| JB | 14 | 669 | 1,557,121 | 198 | 3,437 |  |  | 316 | 6,175 | **59** | **957** |
| JN | 22 | 488 | 1,403,155 | 476 | 7,911 |  |  | 184 | 3,465 | **45** | **825** |
| GB | 23 | 620 | 1,643,434 | 240 | 4,674 |  |  | 170 | 3,389 | **57** | **1,039** |
| GN | 18 | 565 | 1,281,401 | 172 | 3,145 |  |  | 217 | 4055 | **49** | **928** |
| JJ | 2 | 251 | 542,822 | 33 | 570 |  |  | 244 | 3974 | **22** | **418** |
| Total | 154 | 5,122 | 12,646,088 | 1,911 | 33,582 |  |  | 2169 | 41,154 | **467** | **8,254** |

*Sero-monitoring post-vaccination in pigs was not carried out in May (indicated grey color).

|  |  |  |  | **Goats** | | | | | | | |
| --- | --- | --- | --- | --- | --- | --- | --- | --- | --- | --- | --- |
| Province | Total districts in province | Total number of farms | Cattle  Population | Total number of sampled | | | | | | | |
|  |  |  |  | April | | **May** | | October | | **November** | |
|  |  |  |  | Farm | Animal | **Farm** | **Animal** | Farm | Animal | **Farm** | **Animal** |
| GG | 31 | 982 | 33,880 | 2 | 10 | **17** | **85** | 14 | 81 | **15** | **119** |
| GW | 18 | 1,005 | 27,649 | 3 | 15 | **16** | **80** | 21 | 105 | **16** | **80** |
| CB | 11 | 1,914 | 75,466 | 5 | 25 | **40** | **200** | 7 | 35 | **33** | **165** |
| CN | 15 | 2,065 | 54,604 | 8 | 40 | **35** | **175** | 7 | 40 | **31** | **155** |
| JB | 14 | 1,562 | 89,614 | 7 | 35 | **48** | **240** | 3 | 15 | **32** | **160** |
| JN | 22 | 2,376 | 116,998 | 8 | 40 | **60** | **300** | 12 | 60 | **46** | **230** |
| GB | 23 | 2,210 | 73,347 | 8 | 40 | **44** | **220** | 0 | 0 | **36** | **180** |
| GN | 18 | 2,774 | 48,380 | 19 | 105 | **46** | **230** | 14 | 106 | **43** | **215** |
| JJ | 2 | 52 | 5,318 | 0 | 0 | **4** | **20** | 0 | 0 | **2** | **10** |
| Total | 154 | 14,940 | 525,926 | 60 | 310 | **310** | **1,550** | 78 | 442 | **254** | **1,314** |

| Test-kit | PrioCHECK^TM^ FMDV  Type O Ab strip kit | BIONOTE FMD Type O Ab ELISA | VDPro® FMDV Type O Ab  b-ELISA |
| --- | --- | --- | --- |
| **Country/Supplier** | Netherlands/  Thermo Fisher Scientific | Korea/  BIONOTE Inc., | Korea/  Median Diagnostics |
| **ELISA format** | Blocking ELISA | Competitive ELISA | Blocking ELISA |
| **Antigen** | Inactivated virus  (O1 Manisa, ME-SA)  coated with trapping mAb^a^ | Recombinant VP4231  proteins (O/SKR/JC/2014, SEA)  coated | Recombinant P13C  proteins (O/SKR/CJ/2000, ME-SA) coated with trapping mAb |
| **Sample volume** | 20uL | 25uL | 20uL |
| **Detector** | mAb a-FMD Type O-HRP conj. | mAb a-FMD Type O-HRP conj | mAb a-FMD Type O-HRP conj |
| **Calculation** | PI = 100-(O.D_sample_ /O.D_max_) X 100 | PI = 100-(O.D_sample_ /O.D_max_) X 100 | S/N = O.D _sample_ /O.D_NC_ |
| **Cut-off value*** | PI ^b^≥ 50% | PI ≥ 50% | S/N^c^ ≤ 0.6 |
| **Sensitivity**** | (Cattle) 95.2 %, (Pigs) 81.4 % | (Cattle) 100.0 %, (Pigs) 83.2 % | (Cattle) 98.3 %, (Pigs) 91.8 % |
| **Specificity**** | (Cattle) 96.9 %, (Pigs) 97.1 % | (Cattle) 90.3 %, (Pigs) 86.8 % | (Cattle) 93.0 %, (Pigs) 84.7 % |

**Supplementary Table 3. Summary of commercial FMDV Type-O ELISAs used for sero-monitoring post-vaccination**

^a^ Monoclonal antibody, ^b^ Percentage of inhibition, ^c^ Sample to negative ratio

^*^ Interpretation of the result: positive (PI≥50%, S/N≤0.6) or negative (PI<50%, S/N>0.6)

^**^ Validated data for three ELISAs were presented in EUFMD OS20 Poster section (Kwon *et al.*) using 517 samples (290 seropositives/ 227 seronegitives ) for cattle and1, 524 samples for pigs (1,282 seropositives / 242 seronegatives)

| Test-kit | BIONOTE FMD  NSP Ab ELISA | VDPro® FMDV  NSP Ab ELISA |
| --- | --- | --- |
| Country/Supplier | Korea/ Bionote | Korea/ Median Diagnostics |
| ELISA format | Competitive ELISA (Ag capture) | Blocking ELISA (Ag coated) |
| Antigen | 3ABC/*baculovirus* | 3AB/*E.coli* (GST fusion) |
| Sample amount | 50uL | 20uL |
| Detector | Anti 3B mAb^a^-HRP conj. | Anti 3B mAb-HRP conj. |
| Calculation | PI = 100-(O.D_sample_ /O.D_max_) X 100 | S/N = O.D _sample_ /O.D_NC_ |
| Cut-off value^*^ | PI^b^  ≥ 50% | S/N^c^ ≤ 0.6 |

**Supplementary Table 4. Summary of commercial FMD NSP ELISAs used for sero-monitoring post-vaccination**

^a^ Monoclonal antibody, ^b^ Percentage of inhibition, ^c^ Sample to negative ratio

^*^ Interpretation of the result: positive (PI≥50%, S/N≤0.6) or negative (PI<50%, S/N>0.6)
